# Health resource utilization and cost analysis in Medicare beneficiaries with chronic wounds: outcomes with porcine placental extracellular matrix versus standard of care and other advanced treatments

**DOI:** 10.57264/cer-2026-0063

**Published:** 2026-08-18

**Authors:** Jenny Levinson, Janet Mackenzie, Rebecca Decker, Serena Nally, Irene Varghese, Caitlin Sheetz, Peter Kardel, Cristin Taylor

**Affiliations:** 1 Convatec Ltd, Lexington, MA 02421, USA; 2 ADVI Health LLC, Washington, DC 20004, USA

**Keywords:** CAMPS, cellular acellular matrix-like products, Center for Medicare and Medicaid Services, CMS, DFU, diabetic foot ulcer, diabetic ulcer, ECM, extracellular matrix, hard-to-heal wounds, health economics, retrospective study, venous leg ulcer, VLU, wound care

## Abstract

**Aim:**

To compare the healthcare resource utilization, spending and clinical outcomes associated with porcine placental extracellular matrix (PPECM [InnovaMatrix^®^ AC, Convatec Triad Life Sciences, LLC, TN, USA]) versus standard of care (SOC) and other advanced treatments (AT) among Medicare Fee-For-Service beneficiaries with diabetic foot ulcers (DFU) or venous leg ulcers (VLU).

**Materials & methods:**

A retrospective cohort study was conducted using 100% Medicare Parts A and B administrative claims from October 2021 through December 2024. Treatment episodes for DFU and VLU were identified and categorized as PPECM plus SOC, SOC alone or AT plus SOC. Patients were followed for 6 months after the end of each treatment episode. Outcomes included post-episode healthcare utilization by site of care, per-patient-per-month spending, wound-related complications and amputations.

**Results:**

The final study sample included 109,786 eligible DFU patients (117,633 episodes of care; PPECM 0.2%, SOC 95.0%, AT 4.8%) and 53,624 eligible VLU patients (56,498 episodes of care; PPECM 0.1%, SOC 95.5%, AT 4.4%). In DFU, PPECM was associated with significantly lower post-episode utilization in most sites of care compared with SOC, and fewer wound complications including amputations compared with AT. Although total post-episode costs were higher for PPECM than SOC and AT in DFU, PPECM had lower overall cost at specific types of care sites. In VLU, PPECM was associated with significantly fewer visits across most sites of care compared with AT and SOC. PPECM-treated VLU episodes had significantly lower total costs than both SOC and AT. Rates of wound complications and amputations were similar across treatment groups in VLU.

**Conclusion:**

Treatment with PPECM demonstrated lower overall post-episode costs compared with SOC and other AT while maintaining safety outcomes, particularly in VLU, suggesting potential value in chronic wound management.

## Plain language summary: Comparison of healthcare utilization and spending of porcine placental extracellular matrix versus standard of care and other advanced treatments in the treatment of diabetic foot ulcers and venous leg ulcers in the Medicare population

**What is this article about?:**

Diabetic foot ulcers and venous leg ulcers are often hard-to-heal wounds associated with high healthcare costs and health risks, such as infection, amputation and hospitalization. This study compares healthcare utilization and outcomes among Medicare beneficiaries with these hard-to-heal wounds treated with porcine placental extracellular matrix (PPECM) versus other treatments.

**What were the results?:**

PPECM was associated with comparable safety outcomes to standard of care and other advanced treatments. In patients with venous leg ulcers, it was associated with lower overall costs post-wound episode, and in those with diabetic foot ulcers, it was associated with fewer wound complications/amputations.

**What do the results mean?:**

PPECM demonstrated comparable or favorable healthcare utilization and clinical performance across treatment settings compared with standard of care and other advanced treatments for Medicare beneficiaries with hard-to-heal wounds, which supports its potential value in chronic wound management.

Diabetic foot ulcers (DFU) and venous leg ulcers (VLU) are wounds that are often difficult to heal and pose a significant clinical and economic burden to patients [1,2]. Patients with DFU or VLU experience pain, inflammation, discharge and loss of function. In the US, it is estimated that each year up to 3,000,000 and 600,000 people are affected by DFU and VLU, respectively [3,4].

Appropriate treatments include wound care, surgical removal of infected and necrotic tissue, appropriate wound dressings and more advanced treatments (AT) like leg bypass surgery or other vascular surgical procedures for some cases [1,5]. Prompt treatment of DFU and VLU is crucial as patients are at risk of severe complications, infection, amputation and in severe cases death. However, only 30–40% of DFU and about 60% of VLU heal at 3 months of treatment [5,6]. Up to 60% of DFUs become infected, with up to 20% of infections resulting in amputations [5]. Within 5 years of DFU diagnosis, reported mortality rates range from nearly 50% to up to 90% in patients with a major amputation [7].

In addition to substantial morbidity and mortality, DFU and VLU are associated with a high economic burden. In high-income countries, hard-to-heal wounds account for up to 3% of total healthcare expenditures. In 2019, cost projections for all wounds among Medicare beneficiaries ranged from $22.5–67 billion, and up to 50% of wounds are classified as hard-to-heal [4]. In the US, the cost to treat an advanced stage wound episode typically surpasses $50,000 [5,8].

Skin substitutes are advanced therapies designed to accelerate wound healing and reduce complications in non-healing ulcers [9]. They may serve as temporary or permanent coverage to support tissue regeneration, provide a protective environment, reduce infection risk and aid in overall wound healing. Skin substitutes have been widely accepted as the standard of care (SOC) for treating refractory DFU and VLU [10].

Cellular, acellular and matrix-like products (CAMPs) represent a broad class of biomaterials, synthetic materials or biosynthetic matrices designed to treat wounds that do not improve despite SOC treatment [9]. Porcine placental extracellular matrix (PPECM, InnovaMatrix^®^ AC) is the first CAMP derived from porcine placenta that is cleared by the US FDA for wound management, including DFU and VLU. Studies have shown CAMPs to be more effective than SOC in treating patients with DFU [11,12]. A recent study evaluated the use of PPECM in older adults with refractory wounds, 52% of which were classified as limb/life threatening. This analysis demonstrated that 53.3% of patients treated with PPECM achieved wound closure in a median of 53 days, despite previously showing clinical stagnation after SOC treatment [13]. Randomized controlled trials comparing extracellular matrix (ECM) to SOC in VLU found higher rates of healing among patients treated with ECM with or without SOC (55–80%) than SOC alone (24–65%) at 8–12 weeks [14–16]. Systematic reviews of RCTs have found that compared with SOC, treatment with CAMPs for DFU and VLU is associated with improved and accelerated wound healing [17,18]. Similarly, a recent retrospective chart review found that among patients with DFU or VLU, those treated with CAMP achieved a 55% higher rate of full healing at 12 weeks compared with SOC (collagen graft), with a 50% and 95% reduction in time to heal DFU and VLU, respectively [19].

Recent studies investigating healthcare resource utilization and costs among Medicare beneficiaries have demonstrated that treatment with CAMPs versus SOC for DFU and VLU are associated with significant reductions in amputations, emergency department visits and hospital readmissions [3,6]. A recent study also found that compared with other CAMPs, PPECM was associated with significantly fewer outpatient amputations, bacteremia episodes and hospital visits among patients with DFU [7].

This study aims to compare healthcare resource utilization, spending, and clinical outcomes associated with PPECM versus SOC and other AT among Medicare Fee-For-Service (FFS) beneficiaries with DFU or VLU.

## Materials & methods

### Data source & study design

**Figure 1.**
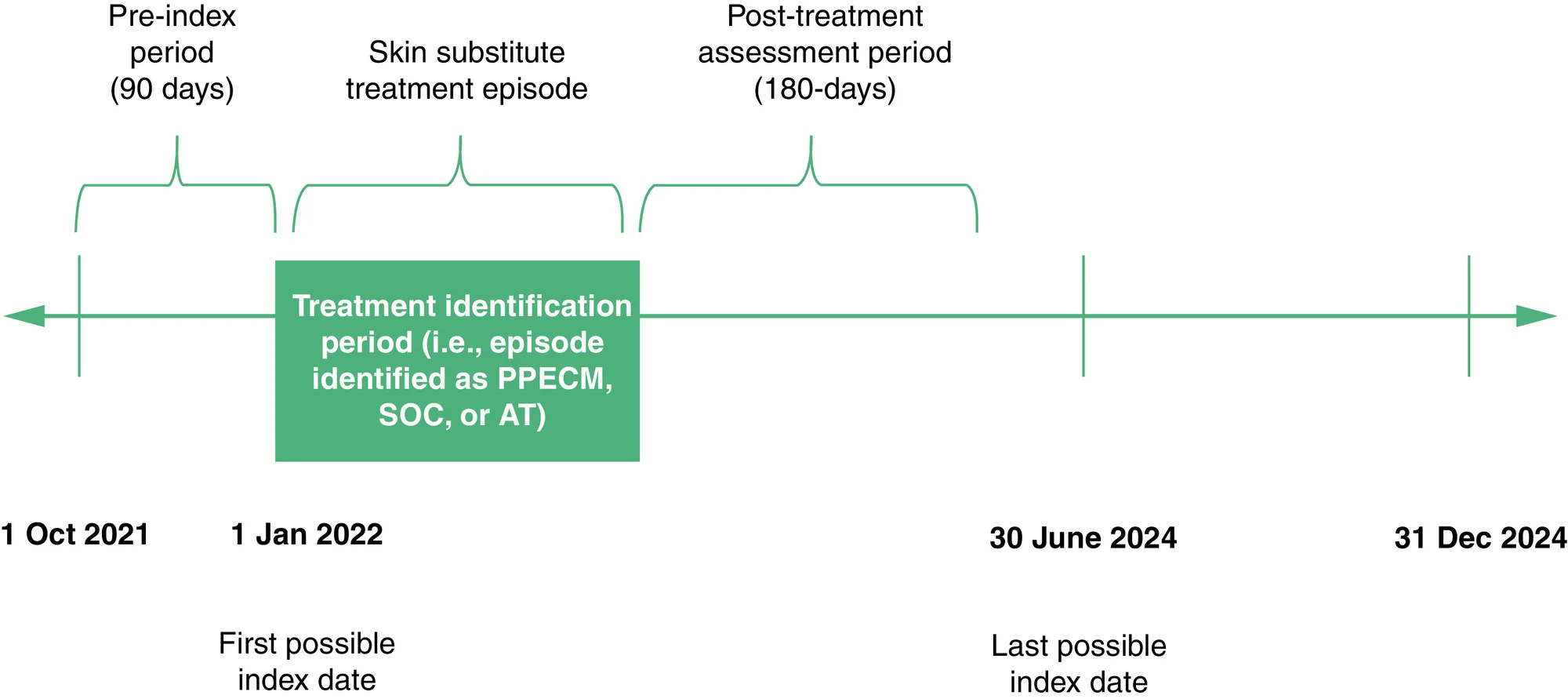
Study design. AT: Other advanced treatment; PPECM: Porcine placental extracellular matrix; SOC: Standard of care.

A comprehensive retrospective cohort study was employed to investigate the utilization patterns of PPECM compared with SOC and other AT competitors in Medicare FFS administrative claims database. DFU and VLU treatment episodes were identified (see below for definition) and designated as either using PPECM (InnovaMatrix^®^ AC, Convatec Triad Life Sciences, LLC, Memphis, TN, USA) plus SOC, SOC alone (Surgical debridement, total contact casting, compression, non-surgical selective debridement and dressing changes, general debridement, SOC-dressing) or AT (Collagen dressings, platelet-rich plasma, negative pressure wound treatment, electrostimulation, MIST therapy, hyperbaric oxygen, topical oxygen) plus SOC. Upon conclusion of the treatment episodes, patients were followed for a 6-month period after the end of their treatment episode. Patients could have multiple treatment episodes if the evaluation periods were not overlapping. Figure 1 provides a summary of the study design.

#### Data source

This study utilized multiple administrative claims datasets from the Centers for Medicare and Medicaid Services (CMS) for the evaluation of the Medicare FFS population. These datasets included: 100% Parts A and B claims from the Research Identifiable Files (RIF) and the Master Beneficiary Summary File (MBSF). These datasets provide 100% of the patients’ utilization, spending, enrollment information and patient demographics. The period assessed was from October 2021 through December 2024. Patients with incomplete demographics or claims data were excluded from the study. The data were accessed by ADVI Health, LLC, who maintain a data use agreement with CMS to access the RIFs and MBSFs through the Virtual Research Data Center.

### Patient population selection

Medicare Parts A and B claims were reviewed for relevant International Statistical Classification of Disease and Related Health Problems (ICD-10-CM) diagnoses to first identify patients with DFU or VLU.

#### DFU treatment episode selection criteria

Patients were required to have an applicable diagnosis of DFU (Appendix Table B) and non-chronic ulcer (ICD-10 Dx L97.x) on the same day to ensure that the patients were purely treated for DFU. Then patients were excluded if the diagnosis code included other parts of the leg (i.e., thigh [L97.1x], calf [L97.2x], ankle [L97.3x]). Patients were ensured to be newly diagnosed by no ulcer diagnosis being present 90 days prior to the initial diagnosis.

#### VLU treatment episode selection criteria

Patients were required to have an applicable diagnosis of VLU (Appendix Table C) and non-chronic ulcer (ICD-10 Dx L97.x) on the same day. Again, to ensure that the patients were purely treated for VLU, patients were excluded if diagnosis codes for treatment included other regions of the leg (i.e., thigh [L97.1x], heel [L97.4x], ‘other part of the foot’ [L97.5]) were present. Patients were also excluded if they had additional confounding diagnoses such as squamous cell carcinoma (C44.xx), leprosy (A30.xx), cutaneous mycobacterial infection (A31.1x), leishmaniasis (B55.x) and gangrenous pyoderma (L88.x). Additionally, patients were ensured to be newly diagnosed by confirming there was no ulcer diagnosis present 90 days prior to the initial diagnosis.

#### Post treatment episode selection criteria & group identification

After the initial diagnosis of DFU and VLU, patients were then assessed for the treatments of interest (i.e., PPECM, SOC or AT) as identified by relevant Category 1 and Category 2 HCPCS codes which are summarized in Appendix Table A. All subsequent treatment was consolidated into an episode of treatment until there was a 90-day gap in treatment. Additional treatments following a 90-day gap were counted as a new episode.

All treatment episodes were assigned to a treatment group. Patients receiving PPECM and AT during the same episode were excluded from the analysis. However, PPECM and AT cohorts could include SOC identifiers. Therefore, the PPECM and AT are also inclusive of SOC, and the SOC cohort does not contain either PPECM or AT codes. Additionally, patients were excluded from the analysis if they had a diagnosis of wound with a depth to the bone (i.e., ICD-10 diagnosis codes of L97.xx4 and L97.xx6) in order to exclude extreme treatment.

Patients were required to have continuous Medicare FFS coverage in Parts A and B starting 90 days prior to their initial diagnosis and continuing 6-months after the conclusion of the treatment episode.

### Study measures

Patient demographics measured at the first diagnosis date included age, gender, race, dual enrollment in Medicare and Medicaid, current reason for Medicare entitlement and comorbidities. All covariates were evaluated for differences to ensure accurate comparisons between treatment groups (see ‘Statistical Analysis’ for information on covariates balancing).

Additionally, baseline clinical characteristics included the Charlson Comorbidity Index (CCI) as well as wound depth (assessed via ICD-10-CM codes L97.xxx) and wound location (assessed via ICD-10-CM codes L97.2x [calf], L92.3x [ankle], L97.4x [heal/mid-foot], L97.5x [other part of foot], L97.8x [lower leg], L97.9x [unspecified lower leg]).

### Statistical analysis

Descriptive statistics were used to summarize the demographics and baseline characteristics of the study population. Categorical variables were reported using percentages, and continuous variables were reported using mean and standard deviation. Comparisons were made of both categorical and continuous variables, using chi-square and *t*-test, respectively. Differences in treatment groups were presented in terms of p-values and standard mean differences (SMD). Due to the correlation between p-values and population size, the SMD was used to determine imbalance, with SMD ≥10 indicating an imbalanced variable between groups. Medicare payment amounts were described on a per-patient-per-month basis. Per-patient-per-month during the study period was expressed as monthly incidence, calculated as the total number of events divided by total follow-up months. To assess differences between groups after inverse probability of treatment weighting (IPTW), weighted chi-square tests were performed to assess categorical variables for independence. For continuous variables, weighted *t*-tests were employed to evaluate differences in means between the groups. Regression models were used to further evaluate outcomes and control data skewness. Poisson regression models were used for patient visits and gamma regressions were used to determine patient cost by place of service. Differences with values of 0.05 or less were considered statistically significant. All analyses were completed using SAS software v.9.4 (SAS Institute Inc, Cary, NC, USA).

#### Cohort balancing with IPTW

IPTW was performed to ensure balance in characteristics and control for differences between treatment groups. The IPTW was independently applied to DFU patients and VLU patients to ensure that the comparison cohorts within each indication were balanced with each other, rather than across the indications. The minimum number of covariates were applied in the IPTW to achieve no SMD ≥10 for key demographic and clinic variables to ensure minimal manipulation of the analytic file. Selected covariates used in the IPTW are summarized within the Results.

## Results

### Patient population selection

From January 2021 to June 2024, a total of 520,131 patients in the Medicare FFS population had a confirmed diagnosis of a DFU and a non-pressure chronic ulcer on the same day. After inclusion and exclusion criteria were applied (see Table 1 for summary), the DFU dataset included 117,633 patient episodes; PPECM made up 0.2% of episodes (n = 225), SOC made up 95.0% (n = 111,707) and AT made up 4.8% (n = 5701).

**Table 1.** Treatment episode selection/attrition – diabetic foot ulcers.

Selection criteria	Unique patient counts	Treatment episodes count
Patients with DFU and applicable diagnosis of non-pressure chronic ulcer diagnosis on same day	520,131	n/a
Without chronic ulcer diagnosis in 90-days prior to first diagnosis	489,110	n/a
Removing patients with exclusion criteria diagnoses (bone depth – L97.xx4 and L97.xx6)	384,228	n/a
Patients with treatments of interest (PPECM, SOC, or AT)	166,856	196,987
Patients with treatments episodes not extending into post-period (i.e., after 1 July 2024)	148,388	171,973
Single treatment type (PPECM, SOC or AT) across episodes	146,613	168,067
Continuously enrolled in Medicare FFS for 90-days prior treatment episode initiation and post-6 months after treatment episode)	109,809	125,590
Removing patients with PPECM and AT combination	109,786	125,567
Patients with complete 6-month applicable episodes	109,786	117,633
Cohort grouping		
PPECM, n (% of total)	225 (0.2%)	225 (0.2%)
SOC, n (% of total)	103,909 (94.6%)	111,707 (95.0%)
AT, n (% of total)	5652 (5.1%)	5701 (4.8%)

AT: Other advanced treatment; DFU: Diabetic foot ulcer; FFS: Fee-for-service; PPECM: Porcine placental extracellular matrix; SOC: Standard of care.

A total of 242,540 patients in the Medicare FFS population had a confirmed diagnosis of a VLU and a non-pressure chronic ulcer on the same day. After inclusion and exclusion criteria were applied (see Table 2 below for summary), the VLU dataset included 56,498 patient episodes; PPECM made up 0.1% of episodes (n = 60), SOC made up 95.5% (n = 53,947) and AT made up 4.4% (n = 2491).

**Table 2.** Treatment episode selection/attrition – venous leg ulcers.

Selection criteria	Unique patient count	Treatment episodes count
Patients with VLU and applicable diagnosis of non-pressure chronic ulcer diagnosis on same day	242,540	n/a
Without chronic ulcer diagnosis in 90-days prior to first diagnosis	222,464	n/a
Removing patients with exclusion criteria diagnoses (squamous cell carcinoma, leprosy, cutaneous mycobacterial infection, leishmaniasis, and pyoderma gangrenous)	182,192	n/a
Patients with treatments of interest (PPECM, SOC or AT)	80,266	91,026
Patients with treatments episodes not extending into post-period (i.e., after 1 July 2024)	70,380	78,449
Single treatment type (PPECM, SOC or AT) across episodes	69,816	77,214
Continuously enrolled in Medicare FFS for 90-days prior treatment episode initiation and post-6 months after treatment episode)	53,634	59,082
Removing patients with PPECM and AT combination	53,624	59,072
Patients with complete 6-month applicable episodes	53,624	56,498
Cohort grouping		
PPECM, n (% of total)	60 (0.1%)	60 (0.1%)
SOC, n (% of total)	51,108 (95.3%)	53,947 (95.5%)
AT, n (% of total)	2456 (4.6%)	2491 (4.4%)

AT: Other advanced treatment; FFS: Fee-for-service; PPECM: Porcine placental extracellular matrix; SOC: Standard of care; VLU: Venous leg ulcer.

### Patient episode characteristics

#### DFU episodes

The three DFU treatment groups were similar across mean age, gender and racial make-up. The mean ages range from 72.0 years (AT) to 73.3 years (SOC). Each group had a higher percentage of males (ranging from 62 to 67%), and the majority were racially White (ranging from 80 to 83%). The proportion of patients with dual eligibility for Medicare/Medicaid (indicating lower income status) was notably lower in the PPECM group (17%) compared with either SOC (27%) or the AT cohort (29%). See Table 3 for additional demographic information.

**Table 3.** Diabetic foot ulcer episode characteristics at baseline for all treatment groups.

Variable	Unweighted cohort					Weighted cohort				
	PPECM, n (%)	SOC, n (%)	SMD between PPECM and SOC	AT	SMD between PPECM and AT	PPECM (%)	SOC, n (%)	SMD between PPECM and SOC	AT, n (%)	SMD between PPECM and AT
Age										
Mean age (st. dev.)	72.38 ± 10.176	73.31 ± 10.888	8.80	71.99 ± 10.393	3.76	73.98	73.25	0.62	73.15	0.69
<Age 65	32 (14%)	18,166 (16%)	5.67	1012 (18%)	9.63	15%	16%	0.37	16%	0.32
Age 65–74	103 (46%)	40,756 (37%)	18.95	2363 (41%)	8.73	37%	37%	0.07	37%	0.02
Age 75–84	70 (31%)	36,820 (33%)	3.96	1727 (30%)	1.77	31%	33%	0.36	33%	0.31
Age ≥85	20 (9%)	15,965 (14%)	16.92	599 (11%)	5.46	17%	14%	0.70	14%	0.68
**Gender**										
Male	150 (67%)	69,593 (62%)	9.13	3612 (63%)	6.94	63%	62%	0.16	62%	0.15
Female	75 (33%)	42,114 (38%)	9.13	2089 (37%)	6.94	37%	38%	0.16	38%	0.15
**Race**										
White	184 (82%)	92,289 (83%)	2.19	4546 (80%)	5.16	80%	82%	0.51	82%	0.41
Black	27 (12%)	10,016 (9%)	9.90	556 (10%)	7.21	9%	9%	0.04	9%	0.01
Asian	* (*)	1222 (1%)	2.18	76 (1%)	0.00	1%	1%	0.24	1%	0.24
Hispanic	* (*)	3161 (3%)	18.88	223 (4%)	23.92	5%	3%	0.77	3%	0.65
Other	* (*)	5019 (5%)	0.24	300 (5%)	3.80	5%	5%	0.02	5%	0.02
**Dual eligibility in Medicare and Medicaid**										
Dual	38 (17%)	30,225 (27%)	24.72	1675 (29%)	29.93	28%	27%	0.23	28%	0.15
Not dual	187 (83%)	81,482 (73%)	24.72	4026 (71%)	29.93	72%	73%	0.23	72%	0.15
**Reason for Medicare enrollment**										
Aged	191 (85%)	92,834 (83%)	4.86	4633 (81%)	9.66	84%	83%	0.25	83%	0.26
Disability	>25 (*)	15,815 (14%)	5.04	856 (15%)	7.47	13%	14%	0.21	14%	0.12
ESRD	* (*)	3058 (3%)	0.44	212 (4%)	5.98	3%	3%	0.13	3%	0.33
**Wound depth**										
L97.xx1 – Non-pressure chronic ulcer of breakdown of skin	27 (12%)	21,083 (19%)	19.09	732 (13%)	2.54	18%	19%	0.16	18%	0.12
L97.xx2 – Non-pressure chronic ulcer of with fat layer exposed	102 (45%)	41,653 (37%)	16.38	1644 (29%)	34.62	40%	37%	0.62	37%	0.59
L97.xx3 – Non-pressure chronic ulcer of necrosis of muscle	* (*)	2708 (2%)	4.50	245 (4%)	14.71	2%	3%	0.13	2%	0.11
L97.xx5 – Non-pressure chronic ulcer of with muscle involvement without evidence of necrosis	* (*)	963 (1%)	5.18	74 (1%)	9.19	<1%	1%	0.58	1%	0.57
L97.xx8 – Non-pressure chronic ulcer of with other specified severity	* (*)	4508 (4%)	10.42	334 (6%)	18.53	4%	4%	0.02	4%	0.03
L97.xx9 – Non-pressure chronic ulcer of with unspecified severity	44 (20%)	23,331 (21%)	3.31	1733 (30%)	25.22	21%	21%	0.15	21%	0.19
Multiple	42 (19%)	17,461 (16%)	8.05	939 (17%)	5.77	14%	16%	0.33	16%	0.30
**Wound location**										
L97.4x – Non-pressure chronic ulcer of heel and midfoot	35 (16%)	14,836 (13%)	6.47	847 (15%)	1.94	15%	13%	0.53	15%	0.07
L97.5x – Non-pressure chronic ulcer of other part of foot	131 (58%)	66,694 (60%)	3.01	3209 (56%)	3.91	54%	60%	1.09	55%	0.27
L97.8x – Non-pressure chronic ulcer of other part of lower leg	21 (9%)	11,765 (11%)	4.00	590 (10%)	3.41	11%	10%	0.20	11%	0.07
L97.9x – Non-pressure chronic ulcer of unspecified part of lower leg	12 (5%)	5044 (5%)	3.78	312 (6%)	0.62	9%	5%	1.48	5%	1.33
Multiple	26 (12%)	13,368 (12%)	1.28	743 (13%)	4.50	11%	12%	0.43	13%	0.64
**Comorbidities**										
Mean CCI (st. dev.)	5.23 ± 2.59	5.406 ± 2.67	6.67	5.62 ± 2.67	14.68	5.65	5.41	0.87	5.53	0.43
Myocardial Infarction	23 (10%)	12,116 (11%)	2.03	703 (12%)	6.66	11%	11%	0.07	12%	0.29
CHF	73 (32%)	39,400 (35%)	5.97	2075 (36%)	8.32	39%	35%	0.76	36%	0.70
Peripheral vascular disease	108 (48%)	47,777 (43%)	10.51	3018 (53%)	9.88	52%	43%	1.86	51%	0.19
Cerebrovascular	30 (13%)	17,376 (16%)	6.32	1066 (19%)	14.65	14%	16%	0.39	18%	1.08
Dementia	16 (7%)	9711 (9%)	5.86	469 (8%)	4.19	7%	9%	0.50	9%	0.43
COPD	45 (20%)	25,856 (23%)	7.65	1381 (24%)	10.18	26%	23%	0.64	24%	0.43
Connective tissue disease	* (*)	5180 (5%)	7.91	284 (5%)	9.50	4%	5%	0.45	5%	0.67
Peptic ulcer disease	0 (0%)	1504 (1%)	16.52	82 (1%)	17.08	0%	1%	–	1%	5.74
Liver disease	11 (5%)	7144 (6%)	6.53	386 (7%)	8.03	5%	6%	0.77	6%	0.72
Diabetes without complications	225 (100%)	111,076 (99%)	10.66	5666 (99%)	11.11	100%	99%	16.16	99%	4.19
Diabetes with complications	179 (80%)	91,725 (82%)	6.49	4657 (82%)	5.39	85%	82%	0.81	81%	1.03
Paraplegia and hemiplegia	* (*)	2837 (3%)	12.74	184 (3%)	16.52	2%	3%	0.49	3%	0.60
Renal disease	97 (43%)	48,340 (43%)	0.33	2604 (46%)	5.16	43%	43%	0.01	44%	0.04
Cancer	25 (11%)	11,575 (10%)	2.42	553 (10%)	4.62	12%	10%	0.51	10%	0.54
Moderate or severe liver disease	* (*)	1141 (1%)	6.76	60 (1%)	7.05	1%	1%	0.41	1%	0.39
Metastatic carcinoma	* (*)	2037 (2%)	0.34	105 (2%)	0.48	3%	2%	0.75	2%	0.72
HIV/AIDS	* (*)	363 (0%)	1.93	14 (0%)	3.39	<1%	<1%	0.31	<1%	0.24

* values less than 11.

Values are presented as mean ± standard deviation or number (%). Standard mean difference (SMD) ≥10 indicates an unbalanced cohort.

AIDS: Acquired immunodeficiency syndrome; AT: Other advanced treatment; CCI: Charlson Comorbidity Index; CHF: Congestive heart failure; COPD: Chronic obstructive pulmonary disease; DFU: Diabetic foot ulcer; ESRD: End-stage renal disease; HIV: Human immunodeficiency virus; PPECM: Porcine placental extracellular matrix; SMD: Standard mean difference; SOC: Standard of care; st. dev.: Standard deviation.

Assessing wound depth at the time of diagnosis found that the PPECM patients had the highest proportion of wounds ‘with the fat layer exposed’ (45%), which was significantly higher than SOC (37%) and AT group (29%). Evaluating wound location found no significant differences between groups.

Lastly, the pre-index period was evaluated for comorbidities with the CCI to help gauge clinical complications and treatment severity. The PPECM group showed no significant differences with the SOC group (mean PPECM CCI = 5.23; SOC CCI = 5.41); however, the AT group was found to have a statistically higher mean CCI (5.62, SMD = 14.68).

Table 3 summarizes the above-mentioned and additional demographic and clinical characteristics for DFU patients.

#### VLU episodes

The PPECM VLU treatment group was significantly older (mean age = 78.3 years) compared with both SOC (76.9) and AT (76.1). However, the groups had similar gender (ranging 45% to 48% male) and racial make-up with the majority being racially White (ranging 85% to 87%). The proportion of patients with dual eligibility for Medicare/Medicaid was significantly lower in the PPECM group (<18% [the exact percentage cannot be shown as it represents counts <11, which is prohibited with the data use agreement with CMS]) compared with the SOC (22%) and AT (23%) groups. Table 4 summarizes VLU patient demographics.

**Table 4.** Venous leg ulcer episode characteristics at baseline for all treatment groups.

Variable	Unweighted cohort					Weighted cohort				
	PPECM, n (%)	SOC, n (%)	SMD between PPECM and SOC	AT	SMD between PPECM and AT	PPECM, n (%)	SOC, n (%)	SMD between PPECM and SOC	AT, n (%)	SMD between PPECM and AT
Age										
Mean age (St Dev)	78.32 ± 9.95	76.94 ± 10.609	13.40	76.06 ± 10.57	21.95	77.55	76.91	0.48	76.84	0.53
<Age 65	* (*)	5035 (9%)	9.81	240 (10%)	10.82	10%	9%	0.12	9%	0.15
Age 65–74	>11 (>20%)	16,309 (30%)	19.56	830 (33%)	26.23	26%	30%	0.83	30%	0.80
Age 75–84	26 (43%)	19,351 (36%)	15.24	898 (36%)	14.86	35%	36%	0.15	36%	0.11
Age ≥85	17 (28%)	13,252 (25%)	8.51	523 (21%)	17.00	29%	24%	0.88	25%	0.78
**Gender**										
Male	28 (47%)	25,887 (48%)	2.63	1126 (45%)	2.93	52%	48%	0.65	44%	1.27
Female	32 (53%)	28,060 (52%)	2.63	1365 (55%)	2.93	48%	52%	0.65	56%	1.27
**Race**										
White	52 (87%)	46,805 (87%)	0.28	2117 (85%)	4.80	80%	87%	1.29	86%	1.10
Black	* (*)	4317 (8%)	6.95	222 (9%)	3.70	13%	8%	1.12	8%	1.01
Asian	* (*)	447 (1%)	7.51	17 (1%)	9.09	4%	1%	1.33	1%	1.39
Hispanic	0 (0%)	651 (1%)	–	45 (2%)	–	0%	1%	–	2%	6.43
Other	* (*)	1727 (3%)	9.94	90 (4%)	12.13	3%	3%	0.17	3%	0.25
**Dual eligibility in Medicare and Medicaid**										
Dual	* (*)	11,682 (22%)	17.20	578 (23%)	20.91	22%	22%	0.01	22%	0.08
Not dual	>48 (>80%)	42,265 (78%)	17.20	(77%)	20.91	78%	78%	0.01	78%	0.08
**Reason for Medicare enrollment**										
Aged	>48 (>90%)	48,813 (91%)	10.43	2242 (90%)	12.03	90%	90%	0.07	90%	0.07
Disability	* (*)	4862 (9%)	8.70	223 (9%)	8.49	10%	9%	0.21	9%	0.32
ESRD	0 (0%)	272 (1%)	–	26 (1%)	–	0%	1%	–	1%	4.87
**Wound depth**										
L97.xx1 – non-pressure chronic ulcer of breakdown of skin	* (*)	8793 (16%)	18.66	331 (13%)	10.23	16%	16%	0.07	16%	0.06
L97.xx2 – non-pressure chronic ulcer of with fat layer exposed	25 (42%)	20,649 (38%)	6.90	935 (38%)	8.42	39%	38%	0.06	38%	0.03
L97.xx3 – non-pressure chronic ulcer of necrosis of muscle	0 (0%)	753 (1%)	–	34 (1%)	–	0%	1%	–	1%	5.75
L97.xx4 – non-pressure chronic ulcer of necrosis of bone	0 (0%)	26 (0%)	–	* (*)	–	0%	0%	–	0%	1.05
L97.xx5 – non-pressure chronic ulcer of with muscle involvement without evidence of necrosis	0 (0%)	324 (1%)	–	37 (2%)	–	0%	1%	–	1%	3.99
L97.xx6 – non-pressure chronic ulcer of with bone involvement without evidence of necrosis	0 (0%)	30 (0%)	–	* (*)	–	0%	0%	–	0%	1.21
L97.xx8 – non-pressure chronic ulcer of with other specified severity	* (*)	2811 (5%)	0.95	131 (5%)	1.17	6%	5%	0.20	5%	0.19
L97.xx9 – non-pressure chronic ulcer of with unspecified severity	16 (27%)	10,849 (20%)	15.46	561 (23%)	9.59	21%	20%	0.16	20%	0.15
Multiple	* (*)	9712 (18%)	3.52	457 (18%)	4.40	18%	18%	0.03	18%	0.06
**Wound Location**										
L97.4x – non-pressure chronic ulcer of heel and midfoot	* (*)	7464 (14%)	17.54	340 (14%)	17.00	10%	14%	1.16	14%	1.12
L97.5x – non-pressure chronic ulcer of other part of foot	11 (18%)	6047 (11%)	20.08	337 (14%)	13.09	9%	11%	0.61	11%	0.63
L97.8x – non-pressure chronic ulcer of other part of lower leg	20 (33%)	22,592 (42%)	17.64	961 (39%)	10.90	44%	42%	0.39	42%	0.38
L97.9x – non-pressure chronic ulcer of unspecified part of lower leg	* (*)	7128 (13%)	9.66	355 (14%)	6.65	15%	13%	0.32	13%	0.32
Multiple	14 (23%)	10,716 (20%)	8.40	498 (20%)	8.08	22%	20%	0.50	20%	0.52
**Comorbidities**										
Mean CCI (St Dev)	3.03 ± 2.46	3.44 ± 2.85	15.15	3.6 ± 2.92	21.06	3.373	3.438	0.20	3.538	0.49
Myocardial infarction	* (*)	3810 (7%)	8.64	196 (8%)	11.67	5%	7%	0.88	8%	1.16
CHF	17 (28%)	20,052 (37%)	18.84	853 (34%)	12.72	27%	37%	1.88	35%	1.39
Peripheral vascular disease	29 (48%)	22,422 (42%)	13.58	1163 (47%)	3.28	50%	42%	1.32	46%	0.63
Cerebrovascular	* (*)	5995 (11%)	3.61	306 (12%)	7.24	7%	11%	1.28	12%	1.57
Dementia	* (*)	3998 (7%)	14.45	198 (8%)	12.46	15%	7%	1.73	8%	1.65
COPD	16 (27%)	13,721 (25%)	2.80	685 (28%)	1.87	27%	25%	0.35	26%	0.17
Connective tissue disease	* (*)	3280 (6%)	2.39	201 (8%)	5.35	8%	6%	0.50	7%	0.11
Peptic ulcer disease	0 (0%)	640 (1%)	–	22 (1%)	–	0%	1%	–	1%	5.01
Liver disease	* (*)	3013 (6%)	4.49	140 (6%)	4.34	5%	6%	0.11	5%	0.04
Diabetes without complications	23 (38%)	21,494 (40%)	3.08	1053 (42%)	8.00	41%	40%	0.15	41%	0.09
Diabetes with complications	14 (23%)	16,583 (31%)	16.67	811 (33%)	20.58	26%	31%	0.93	32%	1.11
Paraplegia and hemiplegia	* (*)	951 (2%)	0.74	69 (3%)	7.47	3%	2%	0.57	2%	0.30
Renal disease	13 (22%)	16,858 (31%)	21.77	783 (31%)	22.17	33%	31%	0.26	31%	0.34
Cancer	* (*)	5237 (10%)	11.07	243 (10%)	11.23	8%	10%	0.39	10%	0.39
Moderate or severe liver disease	* (*)	531 (1%)	5.94	26 (1%)	5.36	2%	1%	0.52	1%	0.48
Metastatic carcinoma	* (*)	904 (2%)	0.07	38 (2%)	1.12	1%	2%	0.34	2%	0.33
HIV/AIDS	0 (0%)	112 (0%)	–	* (*)	–	0%	0%	–	0%	2.37

* values less than 11.

Values are presented as mean ± standard deviation or number (%). Standard mean difference (SMD) ≥10 indicates an unbalanced cohort.

AIDS: Acquired immunodeficiency syndrome; AT: Other advanced treatment; CCI: Charlson Comorbidity Index; CHF: Congestive heart failure; COPD: Chronic obstructive pulmonary disease; ESRD: End-stage renal disease; HIV: Human immunodeficiency virus; PPECM: Porcine placental extracellular matrix; SMD: Standard mean difference; SOC: Standard of care; VLU: Venous leg ulcer.

Wound depth diagnosis codes were similar for the level of ‘with the fat layer exposed’ (ranging from 42 to 38%). PPECM had 27% of patients with a depth of ‘unspecified severity’, which was significantly higher than SOC (20%) but not AT (23%).

With regards to CCI, the PPECM group had a significantly lower mean score (3.03) compared with both SOC (3.44) and AT (3.60).

Table 4 summarizes the above-mentioned and additional demographic and clinical characteristics for VLU patients.

### Balancing cohorts with IPTW

IPTW was used to control for covariates by creating a weighted sample that balanced baseline differences between treatment groups [20], with the goal of emulating the conditions of a randomized control trial where treatment groups are expected to be balanced across key baseline characteristics. Covariates were included in the IPTW model based on clinical relevance and empiric evidence of imbalance, namely a standard mean difference of more than 10 between PPECM and the comparators. Clinical variables were limited to those captured within the claims data (e.g., diagnosis codes and demographic characteristics). Important clinical details including wound severity, factors influencing treatment selection, and expected duration of care are not available in claims data and may contribute to residual confounding. The propensity of receiving PPECM treatment versus the comparators was estimated using multivariate logistic regression applying the covariates (noted below) to calculate a stabilized weighting score for each beneficiary.

For the DFU population, the groups were balanced after controlling for age group (age 65–74), race (Hispanic), dual eligibility for Medicare/Medicaid, wound depth (breakdown of the skin) and peripheral vascular disease. For VLU, the groups were balanced after controlling for age group (age 65–74), dual eligibility for Medicare/Medicaid, reason for Medicare enrollment (aged), wound depth (breakdown of skin), wound location (ulcer of the calf) and chronic heart failure.

Tables 3 & 4 demonstrate that after application of the IPTW methodology (with the ‘weighted cohort’ columns) nearly all variables are balanced in comparison to PPECM, across both indications. The only variable remaining imbalanced was for the DFU cohort, for which the CCI comorbidity of ‘diabetes without complications’ comparison of PPECM and SOC had an SMD = 16.16. However, 100% of PPECM patients had this comorbidity compared with 99.4% of SOC.

### Wound complications

#### DFU wound complications

**Figure 2.**
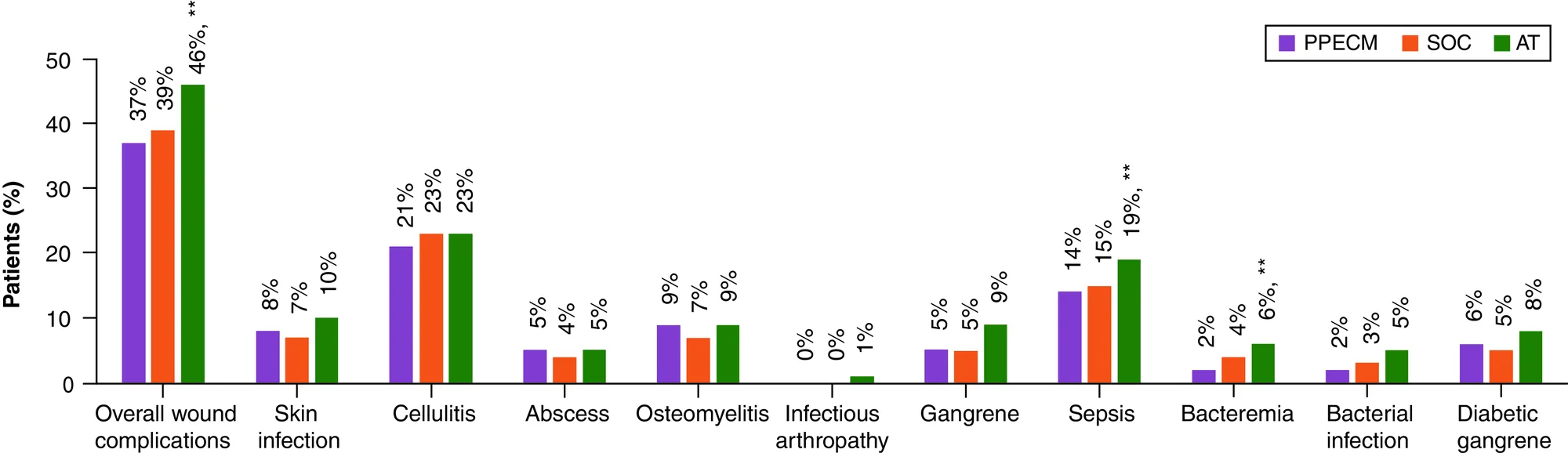
Diabetic foot ulcer wound complications in the 6-month post-episode period. AT: Other advanced treatment; PPECM: Porcine placental extracellular matrix; SOC: Standard of care.

The overall rate of wound complications was assessed in the post-episode period (Figure 2). No significant difference was observed in the total or individual wound complications rates when comparing PPECM to SOC. However, significant differences were noted between the PPECM and AT groups. Across all complications evaluated, the PPECM group (37%) had a lower proportion of patients with wound complications compared with the AT group (46%, p = 0.005). Within individual complications, only bacteremia and sepsis were significantly higher for the AT group compared with PPECM (2% vs 6% [p = 0.003] and 14% vs 19% [p = 0.020], respectively).

#### VLU wound complications

**Figure 3.**
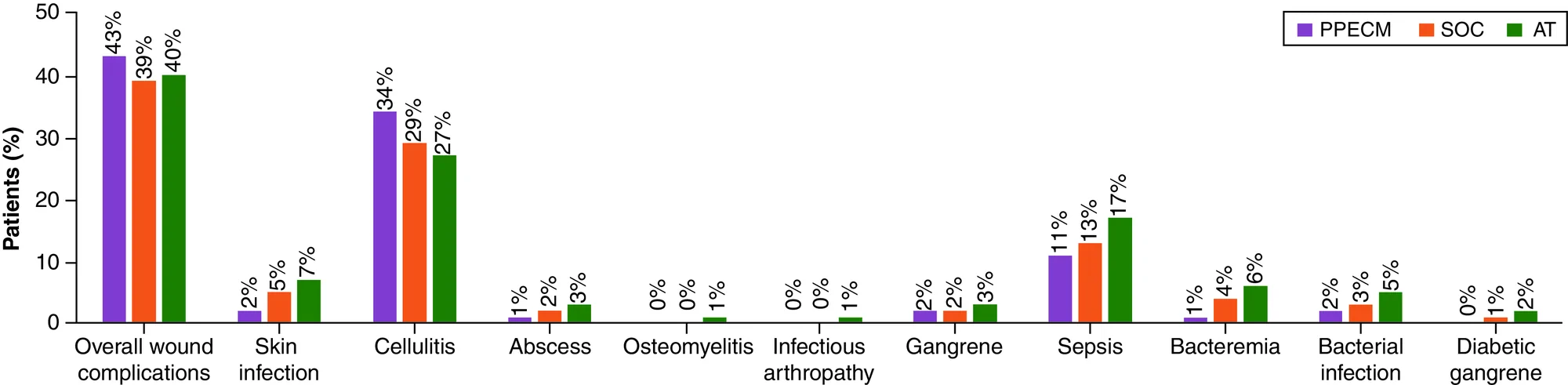
Venous leg ulcer wound complications in the 6-month post-episode period. AT: Other advanced treatments; PPECM: Porcine placental extracellular matrix; SOC: Standard of care.

No significant differences were observed in the total or individual wound complication rates when comparing PPECM to either SOC or AT (Figure 3). PPECM was numerically smaller across all individual types of complications except for cellulitis, but again, the PPECM had non-significant difference between the two groups.

### Amputations in the 6-month post-episode period

**Figure 4.**
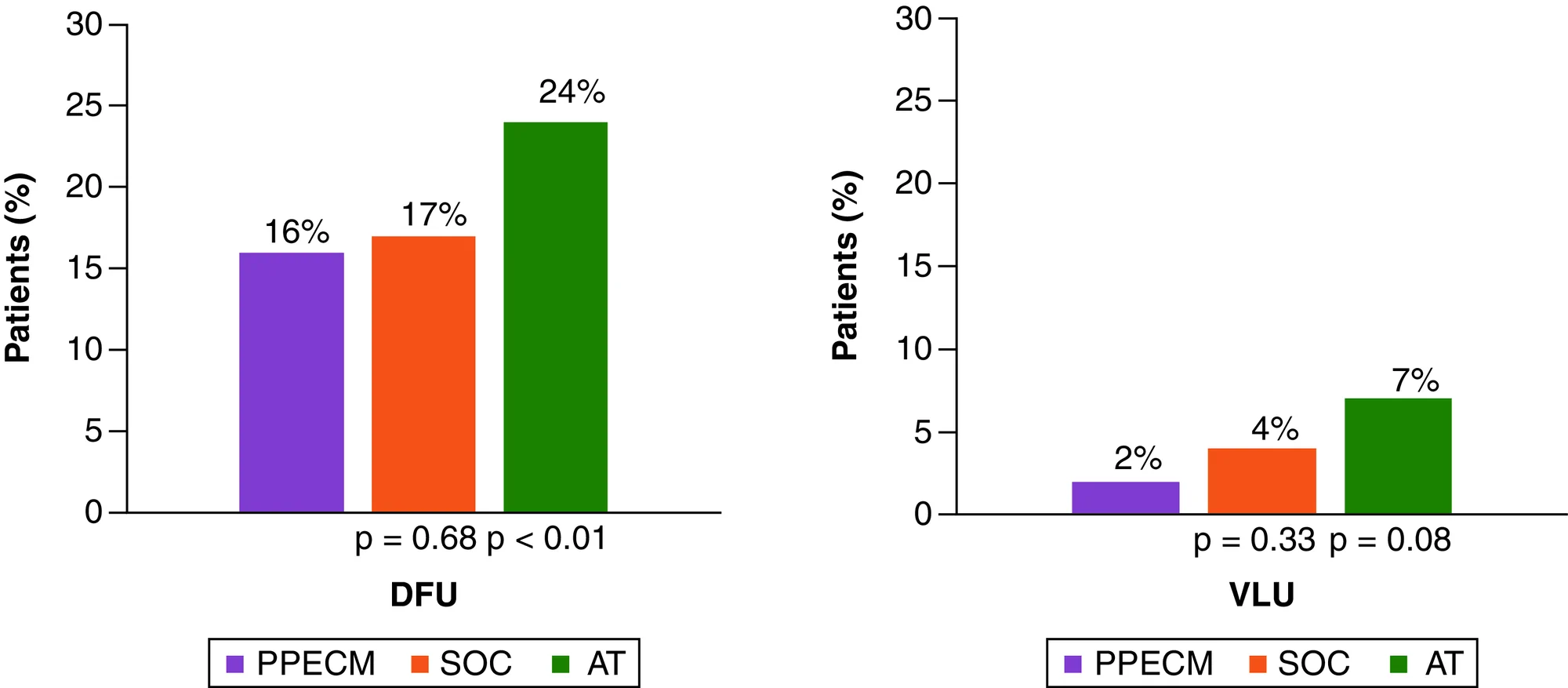
Diabetic foot ulcer and venous leg ulcer major/minor amputations in the 6-month post-episode period. AT: Other advanced treatments; DFU: Diabetic foot ulcers; PPECM: Porcine placental extracellular matrix; SOC: Standard of care; VLU: Venous leg ulcers.

The rate of major and minor amputations (combined) was assessed for inpatient and outpatient claims (Figure 4). For DFU, the PPECM group showed no significant differences with SOC but saw significantly fewer amputations than AT. For VLU, the PPECM group showed no significant differences with SOC or AT; however, while not significant, amputations were substantially lower in PPECM than other AT for VLU (2% vs 7%; p = 0.08).

### Healthcare resource utilization in the 6-month post-episode period

Patients were assessed in the subsequent 6 months after the treatment episode for healthcare utilization in Medicare Parts A and B.

#### DFU utilization comparison

**Figure 5.**
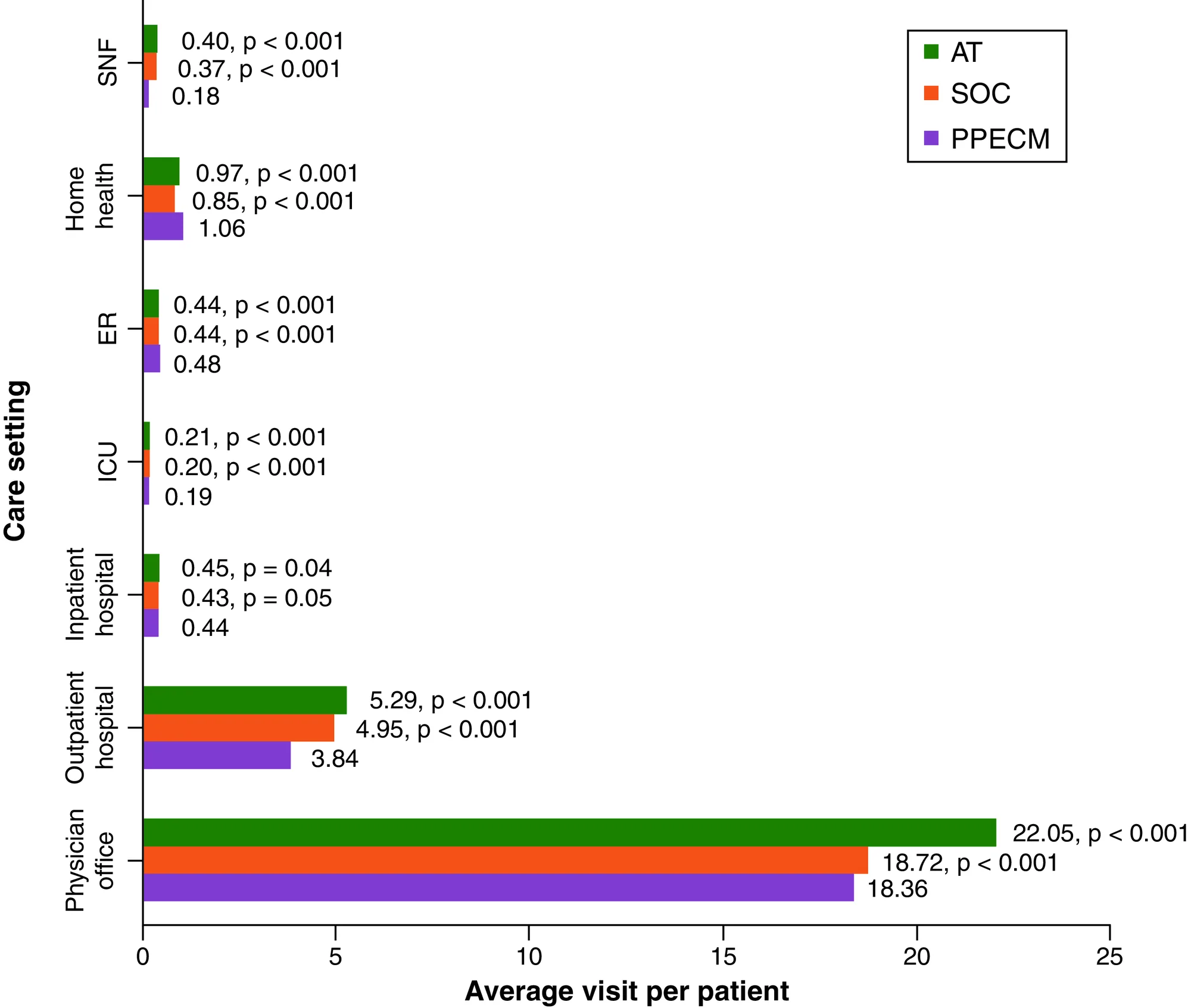
Diabetic foot ulcer cohort average visits per patient in the 6-month post-episode period. AT: Other advanced treatments; ER: Emergency room; ICU: Intensive care unit; PPECM: Porcine placental extracellular matrix; SOC: Standard of care.

Healthcare utilization at sites within Medicare Parts A and B were assessed in the 6-month post-treatment episode period. The point estimates of the Poisson regression found significant differences in utilization between the three treatment groups within the DFU population (Figure 5). The PPECM group had significantly less utilization in the physician office, outpatient hospital, intensive care unit (ICU), and skilled nursing facility (SNF), while having more utilization in emergency room (ER) and home health compared with SOC. PPECM had a similar pattern compared with AT except for inpatient hospital utilization, which was significantly higher for AT.

#### VLU utilization comparison

**Figure 6.**
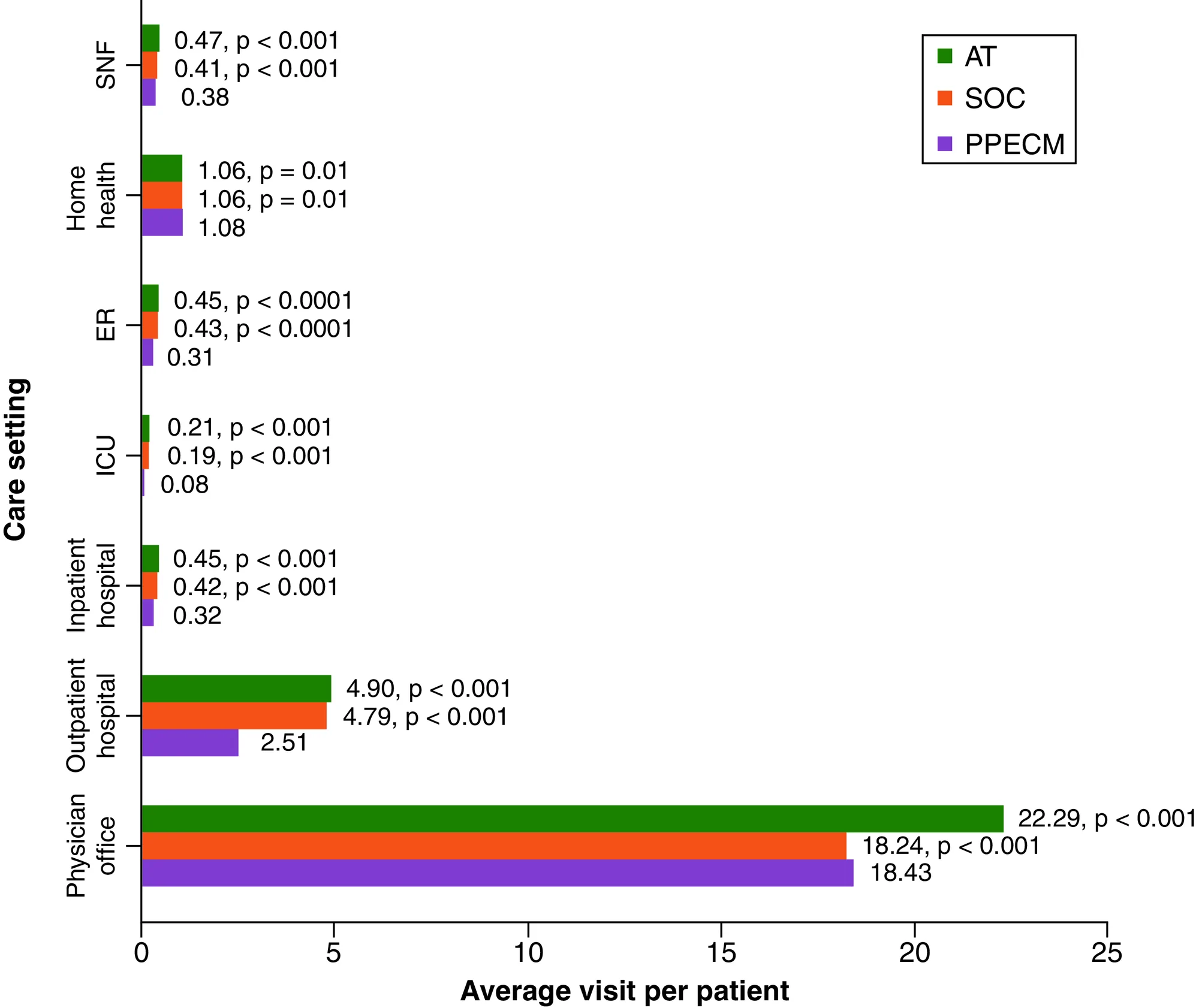
Venous leg ulcer cohort average visits per patient in the 6-month post-episode period. AT: Other advanced treatments; PPECM: Porcine placental extracellular matrix; SNF: Skilled nursing facility; SOC: Standard of care.

The point estimates of the Poisson regression for the VLU cohort also found significant differences in utilization between the three treatment groups (Figure 6). The PPECM group compared with the SOC group had significantly more utilization in the physician office and home health. However, the SOC group had higher utilization in outpatient hospital, inpatient hospital, intensive care unit, ER and SNF. Compared with AT, PPECM had significantly fewer visits across all care settings.

#### Comparison of cohort spending in post period prior to regression

**Figure 7.**
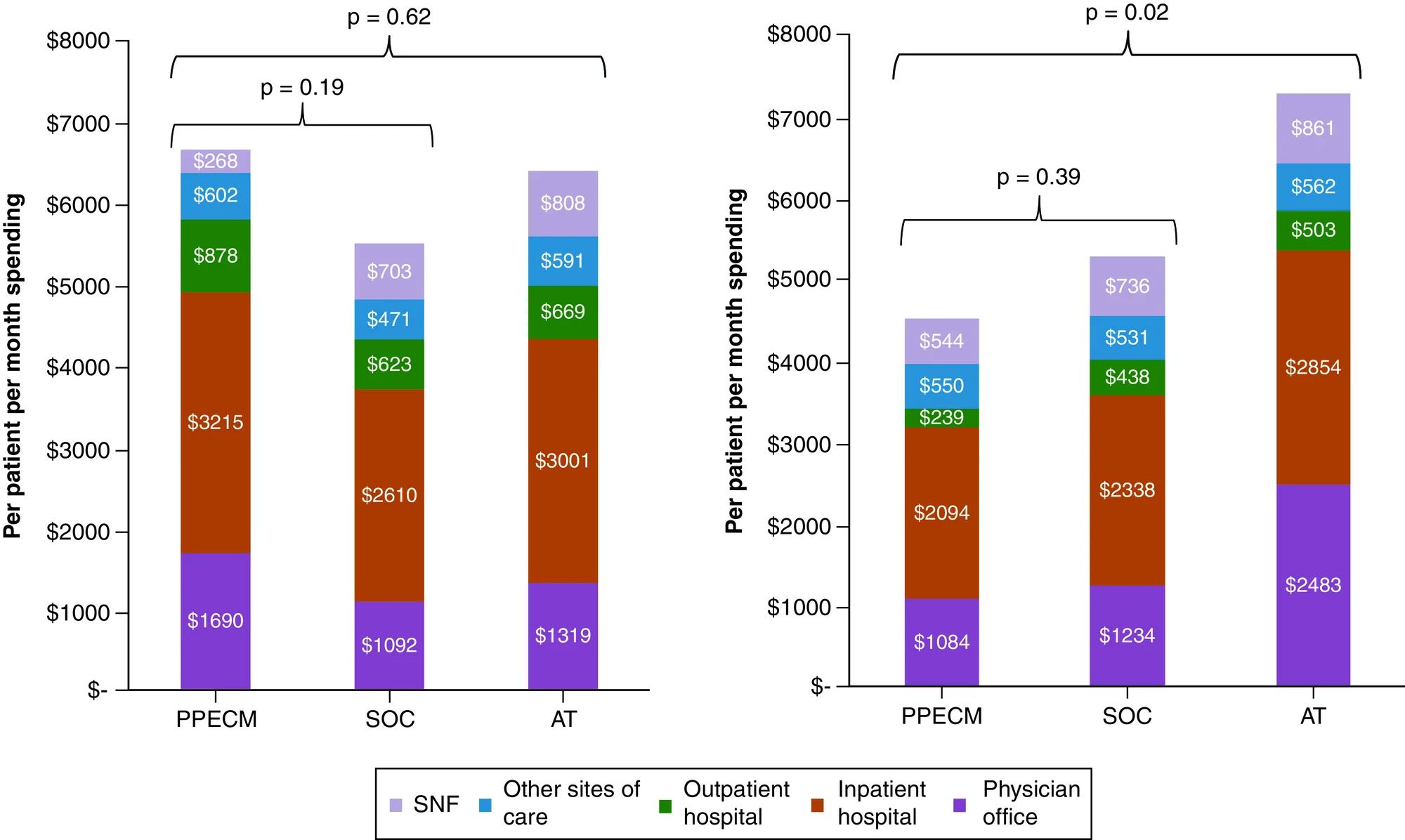
Diabetic foot ulcer (left figure) and venous leg ulcer (right figure) treatment cohorts per patient per month spending in the 6-month post-episode period, by site of care and across sites. AT: Other advanced treatments; PPECM: Porcine placental extracellular matrix; SNF: Skilled nursing facility; SOC: Standard of care.

Before application of regression analyses, total spending was evaluated. For the DFU patients, non-significant differences were found in total spending across all sites of care; however, PPECM had significantly less SNF spend than the two other groups (p < 0.001). For VLU patients, the PPECM group showed no significant difference compared with SOC but was found to have significantly less total spending in the post-treatment period compared with AT (p = 0.02) mainly due to significantly less spending in the physician office and inpatient hospital. Figure 7 summarizes the comparison of PPECM spending by site of care and across sites.

#### Gamma regression point estimates

For DFU, the PPECM patients had significantly higher total cost of care compared with both SOC and AT with most individual sites being higher spend as well (Table 5). Specifically, PPECM was higher in the physician office and outpatient hospital compared with both other groups. For the inpatient setting, PPECM was significantly lower than AT but significantly higher than SOC. In the SNF setting, PPECM had significantly less spend than both groups. With further analysis into the inpatient setting, PPECM had significantly lower spend on medical diagnosis-related groups (DRGs) claims. Additionally, PPECM had significantly lower spend in the inpatient hospital setting compared with AT.

**Table 5.** Diabetic foot ulcer point estimates of gamma regression for 6-month post-episode porcine placental extracellular matrix spending at sites of care.

Site of care	PPECM		SOC			AT		
	Point estimate	95% CI	Point estimate	95% CI	p-value comparison to PPECM	Point estimate	95% CI	p-value comparison to PPECM
Physician office	$1809	[$1786–$1832]	$1158	[$1143–$1173]	<0.0001	$1446	[$1427–$1464]	<0.0001
Outpatient hospital	$1363	[$1341–$1385]	$796	[$785–$808]	<0.0001	$886	[$873–$900]	<0.0001
Inpatient hospital	$9181	[$9030–$9335]	$8555	[$8405–$8708]	<0.0001	$9438	[$9275–$9604]	0.02
Inpatient hospital – medical DRGs	$5495	[$5403–$5588]	$6455	[$6344–$6568]	<0.0001	$7031	[$6911–$7153]	<0.0001
Inpatient hospital – surgical DRGs	$12,215	[$11,898–$12,539]	$9335	[$9057–$9621]	<0.0001	$9458	[$9201–$9722]	<0.0001
DME	$342	[$336–$348]	$224	[$220–$228]	<0.0001	$348	[$342–$354]	0.13
Home health	$1062	[$1051–$1074]	$1152	[$1138–$1167]	<0.0001	$1193	[$1179–$1208]	<0.0001
SNF	$3217	[$3140–$3297]	$5034	[$4941–$5129]	<0.0001	$5499	[$5399–$5600]	<0.0001
Total medical cost (all sites combined)	$7381	[$7286–$7478]	$5952	[$5875–$6029]	<0.0001	$7072	[$6980–$7165]	<0.0001

A p-value less than <0.05 indicates statistical significance.

AT: Other advanced treatments; DFU: Diabetic foot ulcer; DME: Durable medical equipment; DRG: Diagnosis-related group; PPECM: Porcine placental extracellular matrix; SNF: Skilled nursing facility; SOC: Standard of care.

For VLU, the PPECM patients had significantly lower spend in total cost of care compared with both SOC and AT, with lower spend at all sites except for the inpatient hospital (which was significantly higher for PPECM than both groups; see Table 6). When examining the medical/surgical DRGs within the inpatient setting, the PPECM group was significantly higher on medical DRGs (compared with both groups) with no statistical difference on surgical DRGs (compared with both groups).

**Table 6.** Venous leg ulcer point estimates of gamma regression for 6-month post-episode porcine placental extracellular matrix spending at sites of care.

Site of care	PPECM		SOC			AT		
	Point estimate	95% CI	Point estimate	95% CI	p-value comparison to PPECM	Point estimate	95% CI	p-value comparison to PPECM
Physician office	$1114	[$1091–$1137]	$1310	[$1284–$1337]	<0.0001	$2702	[$2647–$2757]	<0.0001
Outpatient hospital	$495	[$482–$508]	$547	[$536–$557]	<0.0001	$667	[$654–$680]	<0.0001
Inpatient hospital	$9448	[$9179–$9725]	$7726	[$7546–$7910]	<0.0001	$8889	[$8688–$9095]	0.001
Inpatient hospital – medical DRGs	$7611	[$7391–$7838]	$6173	[$6027–$6323]	<0.0001	$6942	[$6779–$7110]	<0.0001
Inpatient hospital – surgical DRGs	$9374	[$8907–$9866]	$9204	[$8800–$9626]	0.6	$9136	[$8800–$9486]	0.43
DME	$406	[$395–$418]	$210	[$204–$215]	<0.0001	$284	[$276–$291]	<0.0001
Home health	$1120	[$1101–$1139]	$1188	[$1170–$1206]	<0.0001	$1252	[$1233–$1272]	<0.0001
SNF	$4202	[$4089–$4318]	$4657	[$4550–$4768]	<0.0001	$5243	[$5124–$5365]	<0.0001
Total medical cost (all sites combined)	$4818	[$4724–$4915]	$5786	[$5676–$5898]	<0.0001	$8090	[$7935–$8249]	<0.0001

A p-value less than <0.05 indicates statistical significance.

AT: Other advanced treatments; DME: Durable medical equipment; DRG: Diagnosis-related group; PPECM: Porcine placental extracellular matrix; SNF: Skilled nursing facility; SOC: Standard of care; VLU: Venous leg ulcer.

## Discussion

This retrospective analysis is the first study that investigated healthcare resource utilization and costs associated with PPECM versus SOC and AT in the Medicare FFS population. AT represent a heterogeneous group of therapies that vary in mechanism, composition, and application, with treatment selection influenced by patient complexity, wound characteristics, clinician expertise, site of care and access. As such, observed effects reflect the role of advanced care in real-world practice rather than the performance of individual AT.

PPECM utilization was associated with comparable or favorable clinical and economic outcomes relative to SOC and AT in both the DFU and VLU populations. PPECM was associated with lower overall costs post-wound episode in VLU and fewer wound complications/amputations in DFU.

Across both indications, PPECM was associated with lower rates of healthcare utilization in the 6-month post-treatment period for certain higher-cost sites of care. For example, in DFU, PPECM-treated patients experienced fewer encounters in outpatient hospitals, intensive care units, and skilled nursing facilities, compared with SOC. In VLU, PPECM was associated with lower utilization across most sites of care relative to AT and reduced inpatient and emergency department care compared with SOC. Lower costs in VLU were driven by savings in the acute care setting, which may be suggestive of reduced escalation of care, thereby avoiding high costs associated with these settings without compromising patient outcomes. PPECM was found to reliably have higher rates of (lower cost) home health utilization across ulcer types and comparators. These findings suggest that PPECM may be associated with shifts toward less intensive care following treatment, potentially reflecting the lower rate of complications observed in this study with DFU and reported in the skin substitute literature [3].

Spending outcomes varied by indication. The unadjusted comparison of spending found that the DFU PPECM patients had no significant difference in total cost of care compared with either SOC or AT, whereas the VLU PPECM patients had significantly less spending than AT (with no difference between SOC). For the adjusted analyses, DFU PPECM patients showed higher total cost of care relative to SOC and AT; however, this difference was largely driven by greater physician office and outpatient hospital utilization rather than acute care utilization. In contrast, for VLU patients, PPECM was associated with significantly lower total costs of care compared with both SOC and AT. Cost differences between DFU and VLU cohorts indicate that the economic impact of PPECM is driven, in part, by indication-specific care pathways and healthcare utilization patterns. Differences in the relative reliance on outpatient management versus high-cost acute care settings may underlie the observed cost variation.

Importantly, comparison of rates of negative clinical outcomes found that PPECM was similar or better than the other treatment types. Rates of wound complications and amputations were comparable between PPECM and SOC for both DFU and VLU. However, in DFU, PPECM was found to have significantly fewer wound complications (i.e., lower rates of serious infections such as bacteremia and sepsis) and amputations compared with AT. These findings may explain the higher rates of downstream utilization for the AT group compared with PPECM.

## Limitations

The study examines source data from one payer (Medicare FFS), and the results of the analysis may not be generalizable to other, younger populations (<65 years of age). This study is a retrospective analysis of administrative claims which does not allow for consideration of clinical decisions, uniformity in quality of care by site, patient compliance to care, laboratory values or socioeconomic factors which may influence treatment and outcomes. While IPTW improved balance across observed clinical and demographic variables, important confounders not captured in claims data such as expected treatment duration and factors influencing treatment selection may remain unaccounted for. Product availability by the site of care could potentially bias economic outcomes but was assumed to be similar in this post-market analysis. Despite the robustness of the Medicare FFS dataset, claims data capture only services that are reimbursed, so care paid out-of-pocket or received outside the insurance system may be missed. Interpretation of these findings should consider the imbalance in cohort sizes across treatment groups. The smaller number of PPECM-treated episodes likely reflects its more recent introduction into clinical practice during the study period, which may limit the stability of effect estimates for this group. Although this imbalance may affect generalizability, statistically significant differences between treatment groups were consistently observed across multiple analyses.

## Conclusion

In summary, the results suggest that PPECM represents a beneficial option for refractory ulcers for Medicare beneficiaries with DFU and VLU, with potential benefits in reducing high-intensity healthcare utilization and, in VLU, lowering overall costs of care. While this study is a retrospective observational study, it provides evidence of large-scale, real-world comparisons of PPECM to commonly used treatment strategies.

## Summary points

- Diabetic foot ulcers (DFU) and venous leg ulcers (VLU) are often hard-to-heal wounds that are associated with high rates of morbidity, mortality and healthcare resource utilization.
- The primary objective of this study was to compare healthcare resource utilization, spending, and clinical outcomes associated with porcine placental extracellular matrix (PPECM) versus standard of care (SOC) and other advanced treatments (AT) within the 100% Medicare Research Identifiable Files (October 2021 to December 2024).
- Outcomes of interest included healthcare utilization, total cost of care and clinical outcomes (including rate of amputations and wound complications).
- Patients with diagnoses of DFU or VLU and non-chronic ulcer with at least one medical claim in Medicare Parts A and B were selected from the period between 1 January 2021 to 30 June 2024.
- Eligible patients were assigned to a treatment group: PPECM, SOC or AT.
- Inverse probability of treatment weighting was applied independently to DFU and VLU patients, assessing patient demographics, comorbidities and wound characteristics, to ensure a balanced comparison between treatment groups.
- A total of 117,633 DFU episodes and 56,498 VLU episodes were included in the study; PPECM made up 225 and 60 episodes, respectively.
- In DFU, PPECM was associated with significantly lower post-episode utilization in most sites of care compared with SOC, and fewer wound complications/amputations compared with AT.
- In VLU, PPECM was associated with significantly fewer visits across most sites of care compared with AT and SOC. PPECM-treated VLU episodes had significantly lower total costs than both SOC and AT.
- This retrospective analysis of the Medicare claims database found that patients with DFU or VLU treated with PPECM experienced comparable or favorable clinical and economic outcomes across care settings compared with SOC and AT.

## Supplementary Material


