# Supplementary material for: Health resource utilization and cost analysis in Medicare beneficiaries with chronic wounds: outcomes with porcine placental extracellular matrix versus standard of care and other advanced treatments
Source: J Comp Eff Res. 2026 Aug 18;15(9):e260063. doi: 10.57264/cer-2026-0063 (PMC13556916; doi:10.57264/cer-2026-0063)
Supplement: Supplementary file 1 [file cer-15-260063-s1.docx]

**Appendix Table A. List of Applicable Codes Identifying Treatment Cohorts**

| **Treatment Group** | **Code** | **Code Description** | **Code Type** |
| --- | --- | --- | --- |
| Porcine Placental Extracellular Matrix | A2001 | innovamatrix ac | HCPCS |
| Porcine Placental Extracellular Matrix | A2013 | innovamatrix fs | HCPCS |
| Porcine Placental Extracellular Matrix | A2022 | innovaburn or innovamatrix xl | HCPCS |
| Porcine Placental Extracellular Matrix | A2023 | innovamatrix pd | HCPCS |
| Other Advanced Treatments | A6010 | collagen-based wound filler, dry form, sterile, per gram of collagen. | HCPCS |
| Other Advanced Treatments | A6011 | collagen-based wound filler, gel/paste, per gram of collagen. | HCPCS |
| Other Advanced Treatments | A6021 | collagen dressing, sterile, size 16 sq. in. or less. | HCPCS |
| Other Advanced Treatments | A6022 | collagen dressing, sterile, size more than 16 sq. in. but less than or equal to 48 sq. in. | HCPCS |
| Other Advanced Treatments | A6023 | collagen dressing, sterile, size more than 48 sq. in. | HCPCS |
| Other Advanced Treatments | A6024 | collagen dressing wound filler, sterile, per 6 inches. | HCPCS |
| Other Advanced Treatments | S9055 | procuren or other growth factor preparation to promote wound healing | HCPCS |
| Other Advanced Treatments | P9020 | platelet rich plasma, each unit | HCPCS |
| Other Advanced Treatments | M0076 | prolotherapy | HCPCS |
| Other Advanced Treatments | G0465 | autologous platelet rich plasma (prp) or other blood-derived product for diabetic chronic wounds/ulcers, using an fda-cleared device for this indication, (includes as applicable administration, dressings, phlebotomy, centrifugation or mixing, and all other preparatory procedures, per treatment) | HCPCS |
| Other Advanced Treatments | G0460 | this hcpcs code is used for "autologous platelet rich plasma or other blood-derived product for non-diabetic chronic wounds/ulcers". it includes the phlebotomy, centrifugation, preparation, administration, and dressings related to the prp treatment. under cpt/hcpcs codes group 1: cpt code g0460 has been deleted. this revision is effective for dates of service on or after 8/01/2021. | HCPCS |
| Other Advanced Treatments | 0232T | injection(s), platelet rich plasma, any site, including image guidance, harvesting and preparation when performed | CPT |
| Other Advanced Treatments | 0481T | injection(s), autologous white blood cell concentrate (autologous protein solution), any site, including image guidance, harvesting and preparation, when performed | CPT |
| Other Advanced Treatments | A9272 | wound suction, disposable, includes dressing, all accessories and components, any type, each | HCPCS |
| Other Advanced Treatments | A6550 | wound care set, for negative pressure wound therapy electrical pump, includes all supplies and accessories | HCPCS |
| Other Advanced Treatments | A7000 | canister, disposable, used with suction pump, each | HCPCS |
| Other Advanced Treatments | A9900 | miscellaneous dme supply, accessory and/or service component of another hcpcs code | HCPCS |
| Other Advanced Treatments | E2402 | negative pressure wound therapy electrical pump, stationary or portable | HCPCS |
| Other Advanced Treatments | 97605 | negative pressure wound therapy (e.g., vacuum assisted drainage collection), utilizing durable medical equipment (dme), including topical application(s), wound assessment and instruction(s) for ongoing care, per session, total wound(s) surface area less than or equal to 50 square centimeters. | CPT |
| Other Advanced Treatments | 97606 | negative pressure wound therapy (e.g., vacuum assisted drainage collection), utilizing durable medical equipment (dme), including topical application(s), wound assessment and instruction(s) for ongoing care, per session, total wound(s) surface are greater than 50 square centimeters | CPT |
| Other Advanced Treatments | 97607 | negative pressure wound therapy, (e.g., vacuum assisted drainage collection), utilizing disposable, non-durable medical equipment including provision of exudate management collection system, topical application(s), wound assessment and instructions for ongoing care, per session; total wound(s) surface area less than or equal to 50 square centimeters. | CPT |
| Other Advanced Treatments | 97608 | negative pressure wound therapy, (e.g., pressure wound therapy, (e.g., vacuum assisted drainage collection), utilizing disposable, non-durable medical equipment including provision of exudate management collection system, topical application(s), wound assessment and instructions for ongoing care, per session; total wound(s) surface area greater than 50 square centimeters. | CPT |
| Other Advanced Treatments | 97032 | application of a modality to one or more areas; electrical stimulation (manual), each 15 minutes (not exclusive to this ncd) | CPT |
| Other Advanced Treatments | 97014 | this code covers "electrical stimulation (unattended)" and is commonly used in physical therapy and chiropractic settings for muscle stimulation. | CPT |
| Other Advanced Treatments | E0761 | non-thermal pulsed high frequency radiowaves, high peak power electromagnetic energy treatment device (not covered by medicare) | HCPCS |
| Other Advanced Treatments | E0769 | electrical stimulation or electromagnetic wound treatment device, not otherwise classified | HCPCS |
| Other Advanced Treatments | G0281 | electrical stimulation, (unattended), to one or more areas, for chronic stage iii and stage iv pressure ulcers, arterial ulcers, diabetic ulcers, and venous stasis ulcers not demonstrating measurable signs of healing after 30 days of conventional | HCPCS |
| Other Advanced Treatments | G0282 | electrical stimulation, (unattended), to one or more areas, for wound care other than described in g0281. (not covered by medicare) | HCPCS |
| Other Advanced Treatments | G0283 | this code specifically applies to "electrical stimulation (unattended), wound therapy" and is used by medicare. | HCPCS |
| Other Advanced Treatments | G0295 | electromagnetic stimulation, to one or more areas, for wound care other than described in g0329 or for other uses (not covered by medicare) | HCPCS |
| Other Advanced Treatments | G0329 | electromagnetic therapy, to one or more areas for chronic stage iii and stage iv pressure ulcers, arterial ulcers, diabetic ulcers and venous stasis ulcers not demonstrating measurable signs of healing after 30 days of conventional care as part of a therapy plan of care | HCPCS |
| Other Advanced Treatments | 97610 | low frequency, non-contact, non-thermal ultrasound, including topical application(s), when performed, wound assessment, and instruction(s) for ongoing care, per day | CPT |
| Other Advanced Treatments | 99183 | physician attendance and supervision of hyperbaric oxygen therapy, per session. | CPT |
| Other Advanced Treatments | C1300 | hyperbaric oxygen under pressure, full body chamber, per 30-minute interval. | HCPCS |
| Other Advanced Treatments | A4575 | topical hyperbaric oxygen chamber, disposable | HCPCS |
| Other Advanced Treatments | E0446 | topical oxygen delivery system, not otherwise specified, includes all supplies and accessories | HCPCS |
| Standard of Care | 11000 | debridement of extensive eczematous or infected skin; up to 10% of body surface | CPT |
| Standard of Care | 11001 | debridement of extensive eczematous or infected skin; each additional 10% of the body surface, or part thereof (list separately in addition to code for primary procedure) | CPT |
| Standard of Care | 11042 | debridement, subcutaneous tissue (includes epidermis and dermis, if performed); first 20 sq cm or less | CPT |
| Standard of Care | 11043 | debridement, muscle and/or fascia (includes epidermis, dermis, and subcutaneous tissue, if performed); first 20 sq cm or less | CPT |
| Standard of Care | 11044 | debridement, bone (includes epidermis, dermis, subcutaneous tissue, muscle and/or fascia, if performed); first 20 sq cm or less | CPT |
| Standard of Care | 11045 | debridement, subcutaneous tissue (includes epidermis and dermis, if performed); each additional 20 sq cm, or part thereof (list separately in addition to code for primary procedure) | CPT |
| Standard of Care | 11046 | debridement, muscle and/or fascia (includes epidermis, dermis, and subcutaneous tissue, if performed); each additional 20 sq cm, or part thereof (list separately in addition to code for primary procedure) | CPT |
| Standard of Care | 11047 | debridement, bone (includes epidermis, dermis, subcutaneous tissue, muscle and/or fascia, if performed); each additional 20 sq cm, or part thereof (list separately in addition to code for primary procedure) | CPT |
| Standard of Care | 29445 | application of rigid leg cast | CPT |
| Standard of Care | 29580 | strapping; unna boot | CPT |
| Standard of Care | 29581 | application of multi-layer compression system; leg (below knee), including ankle and foot | CPT |
| Standard of Care | 97597 | debridement (eg, high pressure waterjet with/without suction, sharp selective debridement with scissors, scalpel and forceps), open wound, (eg, fibrin, devitalized epidermis and/or dermis, exudate, debris, biofilm), including topical application(s), wound assessment, use of a whirlpool, when performed and instruction(s) for ongoing care, per session, total wound(s) surface area; first 20 sq cm or less | CPT |
| Standard of Care | 97598 | debridement (eg, high pressure waterjet with/without suction, sharp selective debridement with scissors, scalpel and forceps), open wound, (eg, fibrin, devitalized epidermis and/or dermis, exudate, debris, biofilm), including topical application(s), wound assessment, use of a whirlpool, when performed and instruction(s) for ongoing care, per session, total wound(s) surface area; each additional 20 sq cm, or part thereof (list separately in addition to code for primary procedure) | CPT |
| Standard of Care | 97602 | removal of devitalized tissue from wound(s), non-selective debridement, without anesthesia (eg, wet-to-moist dressings, enzymatic, abrasion, larval therapy), including topical application(s), wound assessment, and instruction(s) for ongoing care, per session | CPT |
| Standard of Care | A6196 | alginate or other fiber gelling dressing, wound cover, sterile, pad size 16 sq. in. or less, each dressing | HCPCS |
| Standard of Care | A6197 | alginate or other fiber gelling dressing, wound cover, sterile, pad size more than 16 sq. in. but less than or equal to 48 sq. in., each dressing | HCPCS |
| Standard of Care | A6198 | alginate or other fiber gelling dressing, wound cover, sterile, pad size more than 48 sq. in., each dressing | HCPCS |
| Standard of Care | A6199 | alginate or other fiber gelling dressing, wound filler, sterile, per 6 inches | HCPCS |
| Standard of Care | A6203 | composite dressing, sterile, pad size 16 sq. in. or less, with any size adhesive border, each dressing | HCPCS |
| Standard of Care | A6204 | composite dressing, sterile, pad size more than 16 sq. in. but less than or equal to 48 sq. in., with any size adhesive border, each dressing | HCPCS |
| Standard of Care | A6205 | composite dressing, sterile, pad size more than 48 sq. in., with any size adhesive border, each dressing | HCPCS |
| Standard of Care | A6206 | contact layer, sterile, 16 sq. in. or less, each dressing | HCPCS |
| Standard of Care | A6207 | contact layer, sterile, more than 16 sq. in. but less than or equal to 48 sq. in., each dressing | HCPCS |
| Standard of Care | A6208 | contact layer, sterile, more than 48 sq. in., each dressing | HCPCS |
| Standard of Care | A6209 | foam dressing, wound cover, sterile, pad size 16 sq. in. or less, without adhesive border, each dressing | HCPCS |
| Standard of Care | A6210 | foam dressing, wound cover, sterile, pad size more than 16 sq. in. but less than or equal to 48 sq. in., without adhesive border, each dressing | HCPCS |
| Standard of Care | A6211 | foam dressing, wound cover, sterile, pad size more than 48 sq. in., without adhesive border, each dressing | HCPCS |
| Standard of Care | A6212 | foam dressing, wound cover, sterile, pad size 16 sq. in. or less, with any size adhesive border, each dressing | HCPCS |
| Standard of Care | A6213 | foam dressing, wound cover, sterile, pad size more than 16 sq. in. but less than or equal to 48 sq. in., with any size adhesive border, each dressing | HCPCS |
| Standard of Care | A6214 | foam dressing, wound cover, sterile, pad size more than 48 sq. in., with any size adhesive border, each dressing | HCPCS |
| Standard of Care | A6215 | foam dressing, wound filler, sterile, per gram | HCPCS |
| Standard of Care | A6216 | gauze, non-impregnated, non-sterile, pad size 16 sq. in. or less, without adhesive border, each dressing | HCPCS |
| Standard of Care | A6217 | gauze, non-impregnated, non-sterile, pad size more than 16 sq. in. but less than or equal to 48 sq. in., without adhesive border, each dressing | HCPCS |
| Standard of Care | A6218 | gauze, non-impregnated, non-sterile, pad size more than 48 sq. in., without adhesive border, each dressing | HCPCS |
| Standard of Care | A6219 | gauze, non-impregnated, sterile, pad size 16 sq. in. or less, with any size adhesive border, each dressing | HCPCS |
| Standard of Care | A6220 | gauze, non-impregnated, sterile, pad size more than 16 sq. in. but less than or equal to 48 sq. in., with any size adhesive border, each dressing | HCPCS |
| Standard of Care | A6221 | gauze, non-impregnated, sterile, pad size more than 48 sq. in., with any size adhesive border, each dressing | HCPCS |
| Standard of Care | A6222 | gauze, impregnated with other than water, normal saline, or hydrogel, sterile, pad size 16 sq. in. or less, without adhesive border, each dressing | HCPCS |
| Standard of Care | A6223 | gauze, impregnated with other than water, normal saline, or hydrogel, sterile, pad size more than 16 sq. in., but less than or equal to 48 sq. in., without adhesive border, each dressing | HCPCS |
| Standard of Care | A6224 | gauze, impregnated with other than water, normal saline, or hydrogel, sterile, pad size more than 48 sq. in., without adhesive border, each dressing | HCPCS |
| Standard of Care | A6228 | gauze, impregnated, water or normal saline, sterile, pad size 16 sq. in. or less, without adhesive border, each dressing | HCPCS |
| Standard of Care | A6229 | gauze, impregnated, water or normal saline, sterile, pad size more than 16 sq. in. but less than or equal to 48 sq. in., without adhesive border, each dressing | HCPCS |
| Standard of Care | A6230 | gauze, impregnated, water or normal saline, sterile, pad size more than 48 sq. in., without adhesive border, each dressing | HCPCS |
| Standard of Care | A6231 | gauze, impregnated, hydrogel, for direct wound contact, sterile, pad size 16 sq. in. or less, each dressing | HCPCS |
| Standard of Care | A6232 | gauze, impregnated, hydrogel, for direct wound contact, sterile, pad size greater than 16 sq. in., but less than or equal to 48 sq. in., each dressing | HCPCS |
| Standard of Care | A6233 | gauze, impregnated, hydrogel, for direct wound contact, sterile, pad size more than 48 sq. in., each dressing | HCPCS |
| Standard of Care | A6234 | hydrocolloid dressing, wound cover, sterile, pad size 16 sq. in. or less, without adhesive border, each dressing | HCPCS |
| Standard of Care | A6235 | hydrocolloid dressing, wound cover, sterile, pad size more than 16 sq. in. but less than or equal to 48 sq. in., without adhesive border, each dressing | HCPCS |
| Standard of Care | A6236 | hydrocolloid dressing, wound cover, sterile, pad size more than 48 sq. in., without adhesive border, each dressing | HCPCS |
| Standard of Care | A6237 | hydrocolloid dressing, wound cover, sterile, pad size 16 sq. in. or less, with any size adhesive border, each dressing | HCPCS |
| Standard of Care | A6238 | hydrocolloid dressing, wound cover, sterile, pad size more than 16 sq. in. but less than or equal to 48 sq. in., with any size adhesive border, each dressing | HCPCS |
| Standard of Care | A6239 | hydrocolloid dressing, wound cover, sterile, pad size more than 48 sq. in., with any size adhesive border, each dressing | HCPCS |
| Standard of Care | A6240 | hydrocolloid dressing, wound filler, paste, sterile, per ounce | HCPCS |
| Standard of Care | A6241 | hydrocolloid dressing, wound filler, dry form, sterile, per gram | HCPCS |
| Standard of Care | A6242 | hydrogel dressing, wound cover, sterile, pad size 16 sq. in. or less, without adhesive border, each dressing | HCPCS |
| Standard of Care | A6243 | hydrogel dressing, wound cover, sterile, pad size more than 16 sq. in. but less than or equal to 48 sq. in., without adhesive border, each dressing | HCPCS |
| Standard of Care | A6244 | hydrogel dressing, wound cover, sterile, pad size more than 48 sq. in., without adhesive border, each dressing | HCPCS |
| Standard of Care | A6245 | hydrogel dressing, wound cover, sterile, pad size 16 sq. in. or less, with any size adhesive border, each dressing | HCPCS |
| Standard of Care | A6246 | hydrogel dressing, wound cover, sterile, pad size more than 16 sq. in. but less than or equal to 48 sq. in., with any size adhesive border, each dressing | HCPCS |
| Standard of Care | A6247 | hydrogel dressing, wound cover, sterile, pad size more than 48 sq. in., with any size adhesive border, each dressing | HCPCS |
| Standard of Care | A6248 | hydrogel dressing, wound filler, gel, per fluid ounce | HCPCS |
| Standard of Care | A6250 | skin sealants, protectants, moisturizers, ointments, any type, any size | HCPCS |
| Standard of Care | A6251 | specialty absorptive dressing, wound cover, sterile, pad size 16 sq. in. or less, without adhesive border, each dressing | HCPCS |
| Standard of Care | A6252 | specialty absorptive dressing, wound cover, sterile, pad size more than 16 sq. in. but less than or equal to 48 sq. in., without adhesive border, each dressing | HCPCS |
| Standard of Care | A6253 | specialty absorptive dressing, wound cover, sterile, pad size more than 48 sq. in., without adhesive border, each dressing | HCPCS |
| Standard of Care | A6254 | specialty absorptive dressing, wound cover, sterile, pad size 16 sq. in. or less, with any size adhesive border, each dressing | HCPCS |
| Standard of Care | A6255 | specialty absorptive dressing, wound cover, sterile, pad size more than 16 sq. in. but less than or equal to 48 sq. in., with any size adhesive border, each dressing | HCPCS |
| Standard of Care | A6256 | specialty absorptive dressing, wound cover, sterile, pad size more than 48 sq. in., with any size adhesive border, each dressing | HCPCS |
| Standard of Care | A6257 | transparent film, sterile, 16 sq. in. or less, each dressing | HCPCS |
| Standard of Care | A6258 | transparent film, sterile, more than 16 sq. in. but less than or equal to 48 sq. in., each dressing | HCPCS |
| Standard of Care | A6259 | transparent film, sterile, more than 48 sq. in., each dressing | HCPCS |
| Standard of Care | A6260 | wound cleansers, any type, any size | HCPCS |
| Standard of Care | A6261 | wound filler, gel/paste, per fluid ounce, not otherwise specified | HCPCS |
| Standard of Care | A6262 | wound filler, dry form, per gram, not otherwise specified | HCPCS |
| Standard of Care | A6266 | gauze, impregnated, other than water, normal saline, or zinc paste, sterile, any width, per linear yard | HCPCS |
| Standard of Care | A6402 | gauze, non-impregnated, sterile, pad size 16 sq. in. or less, without adhesive border, each dressing | HCPCS |
| Standard of Care | A6403 | gauze, non-impregnated, sterile, pad size more than 16 sq. in. less than or equal to 48 sq. in., without adhesive border, each dressing | HCPCS |
| Standard of Care | A6404 | gauze, non-impregnated, sterile, pad size more than 48 sq. in., without adhesive border, each dressing | HCPCS |
| Standard of Care | A6407 | packing strips, non-impregnated, sterile, up to 2 inches in width, per linear yard | HCPCS |
| Standard of Care | A6413 | adhesive bandage, first-aid type, any size, each | HCPCS |
| Standard of Care | A6441 | padding bandage, non-elastic, non-woven/non-knitted, width greater than or equal to three inches and less than five inches, per yard | HCPCS |
| Standard of Care | A6442 | conforming bandage, non-elastic, knitted/woven, non-sterile, width less than three inches, per yard | HCPCS |
| Standard of Care | A6443 | conforming bandage, non-elastic, knitted/woven, non-sterile, width greater than or equal to three inches and less than five inches, per yard | HCPCS |
| Standard of Care | A6444 | conforming bandage, non-elastic, knitted/woven, non-sterile, width greater than or equal to 5 inches, per yard | HCPCS |
| Standard of Care | A6445 | conforming bandage, non-elastic, knitted/woven, sterile, width less than three inches, per yard | HCPCS |
| Standard of Care | A6446 | conforming bandage, non-elastic, knitted/woven, sterile, width greater than or equal to three inches and less than five inches, per yard | HCPCS |
| Standard of Care | A6447 | conforming bandage, non-elastic, knitted/woven, sterile, width greater than or equal to five inches, per yard | HCPCS |
| Standard of Care | A6448 | light compression bandage, elastic, knitted/woven, width less than three inches, per yard | HCPCS |
| Standard of Care | A6449 | light compression bandage, elastic, knitted/woven, width greater than or equal to three inches and less than five inches, per yard | HCPCS |
| Standard of Care | A6450 | light compression bandage, elastic, knitted/woven, width greater than or equal to five inches, per yard | HCPCS |
| Standard of Care | A6451 | moderate compression bandage, elastic, knitted/woven, load resistance of 1.25 to 1.34 foot pounds at 50% maximum stretch, width greater than or equal to three inches and less than five inches, per yard | HCPCS |
| Standard of Care | A6452 | high compression bandage, elastic, knitted/woven, load resistance greater than or equal to 1.35 foot pounds at 50% maximum stretch, width greater than or equal to three inches and less than five inches, per yard | HCPCS |
| Standard of Care | A6453 | self-adherent bandage, elastic, non-knitted/non-woven, width less than three inches, per yard | HCPCS |
| Standard of Care | A6454 | self-adherent bandage, elastic, non-knitted/non-woven, width greater than or equal to three inches and less than five inches, per yard | HCPCS |
| Standard of Care | A6455 | self-adherent bandage, elastic, non-knitted/non-woven, width greater than or equal to five inches, per yard | HCPCS |
| Standard of Care | A6456 | zinc paste impregnated bandage, non-elastic, knitted/woven, width greater than or equal to three inches and less than five inches, per yard | HCPCS |
| Standard of Care | A6457 | tubular dressing with or without elastic, any width, per linear yard | HCPCS |
| Standard of Care | A6531 | gradient compression stocking, below knee, 30-40 mmhg, used as a surgical dressing, each | HCPCS |
| Standard of Care | A6532 | gradient compression stocking, below knee, 40-50 mmhg, used as a surgical dressing, each | HCPCS |
| Standard of Care | A6545 | gradient compression wrap, non-elastic, below knee, 30-50 mmhg, used as a surgical dressing, each | HCPCS |

CPT: Current Procedural Terminology; HCPCS: Healthcare Common Procedure Coding System

**Appendix Table B. Diagnosis Codes Used to Identify DFU**

| **ICD-10-CM Code** | **Code Descriptor** |
| --- | --- |
| E08.621 | Diabetes mellitus due to underlying condition with foot ulcer |
| E08.622 | Diabetes mellitus due to underlying condition with other skin ulcer |
| E09.621 | Drug or chemical induced diabetes mellitus with foot ulcer |
| E09.622 | Drug or chemical induced diabetes mellitus with other skin ulcer |
| E10.621 | Type 1 diabetes mellitus with foot ulcer |
| E10.622 | Type 1 diabetes mellitus with other skin ulcer |
| E11.621 | Type 2 diabetes mellitus with foot ulcer |
| E11.622 | Type 2 diabetes mellitus with other skin ulcer |
| E13.621 | Other specified diabetes mellitus with foot ulcer |
| E13.622 | Other specified diabetes mellitus with other skin ulcer |

DFU: diabetic foot ulcer; ICD-10-CM: International Classification of Diseases, Tenth Revision, Clinical Modification

**Appendix Table C. Diagnosis Code Used to Identify VLU**

| **ICD-10-CM Code** | **Code Descriptor** |
| --- | --- |
| I87.2 | Venous Insufficiency (chronic) (peripheral) |
| I83.011 | Varicose veins of right lower extremity with ulcer of thigh |
| I83.012 | Varicose veins of right lower extremity with ulcer of calf |
| I83.013 | Varicose veins of right lower extremity with ulcer of ankle |
| I83.014 | Varicose veins of right lower extremity with ulcer of heel and midfoot |
| I83.015 | Varicose veins of right lower extremity with ulcer other part of foot |
| I83.018 | Varicose veins of right lower extremity with ulcer other part of lower leg |
| I83.021 | Varicose veins of left lower extremity with ulcer of thigh |
| I83.022 | Varicose veins of left lower extremity with ulcer of calf |
| I83.023 | Varicose veins of left lower extremity with ulcer of ankle |
| I83.024 | Varicose veins of left lower extremity with ulcer of heel and midfoot |
| I83.025 | Varicose veins of left lower extremity with ulcer other part of foot |
| I83.028 | Varicose veins of left lower extremity with ulcer other part of lower leg |
| I83.211 | Varicose veins of right lower extremity with both ulcer of thigh and inflammation |
| I83.212 | Varicose veins of right lower extremity with both ulcer of calf and inflammation |
| I83.213 | Varicose veins of right lower extremity with both ulcer of ankle and inflammation |
| I83.214 | Varicose veins of right lower extremity with both ulcer of heel and midfoot and inflammation |
| I83.215 | Varicose veins of right lower extremity with both ulcer other part of foot and inflammation |
| I83.218 | Varicose veins of right lower extremity with both ulcer of other part of lower extremity and inflammation |
| I83.221 | Varicose veins of left lower extremity with both ulcer of thigh and inflammation |
| I83.222 | Varicose veins of left lower extremity with both ulcer of calf and inflammation |
| I83.223 | Varicose veins of left lower extremity with both ulcer of ankle and inflammation |
| I83.224 | Varicose veins of left lower extremity with both ulcer of heel and midfoot and inflammation |
| I83.225 | Varicose veins of left lower extremity with both ulcer other part of foot and inflammation |
| I83.228 | Varicose veins of left lower extremity with both ulcer of other part of lower extremity and inflammation |
| I87.011 | Postthrombotic syndrome with ulcer of right lower extremity |
| I87.012 | Postthrombotic syndrome with ulcer of left lower extremity |
| I87.013 | Postthrombotic syndrome with ulcer of bilateral lower extremity |
| I87.031 | Postthrombotic syndrome with ulcer and inflammation of right lower extremity |
| I87.032 | Postthrombotic syndrome with ulcer and inflammation of left lower extremity |
| I87.033 | Postthrombotic syndrome with ulcer and inflammation of bilateral lower extremity |
| I87.311 | Chronic venous hypertension (idiopathic) with ulcer of right lower extremity |
| I87.312 | Chronic venous hypertension (idiopathic) with ulcer of left lower extremity |
| I87.313 | Chronic venous hypertension (idiopathic) with ulcer of bilateral lower extremity |
| I87.331 | Chronic venous hypertension (idiopathic) with ulcer and inflammation of right lower extremity |
| I87.332 | Chronic venous hypertension (idiopathic) with ulcer and inflammation of left lower extremity |
| I87.333 | Chronic venous hypertension (idiopathic) with ulcer and inflammation of bilateral lower extremity |

ICD-10-CM: International Classification of Diseases, Tenth Revision, Clinical Modification; VLU: venous leg ulcer
